# Collectanea

**Published:** 1828-01

**Authors:** 


### COLLECTANEA.

Floriferis ut apes in saltibus omnia libant,
Omnia nos, itidem, depascimur aurea dicta.

### PHYSIOLOGY.

*Peculiarities in the Sense of Smell.*—Dr. VOGEL has made some rather interesting remarks on the above subject in HECKER'S, *Litterarische Ann. der gesammten Heilkunde.* Of these we subjoin an abridged account.

The astonishing variety of odours emitted from different bodies is at once a curious and interesting subject of investigation. Not only the animal and vegetable kingdoms, but even stones, earths, and metals, give forth a different scent. Under particular circumstances, also, the same body smells differently at different times. Pure earth and water, ice, glass, the fixed salts, most metals, &c. are generally considered to be inodorous, but they are not so under certain circumstances. Those substances which, to the generality of mankind, appear devoid of scent, are odoriferous to more perfect and acute olfactory organs than they possess. The Peruvian Indians, it is well known, can distinguish, during the night, the various races of Europeans, Americans, or Negroes. The Calmuck can trace his steps through the most extensive heaths by the odour of the various plants upon which he treads in his journey. The traveller from Smyrna or Aleppo to Babylon can determine the distance he is from the latter place by the smell of the sand in the desert.

The sense of smell is most powerful in a state of nature. A boy, who was found wild in the woods of Aveyron, possessed so delicate a sense of smell, that all his powers appeared to be concentrated in the olfactory organ, which in proportion as he became civilised, lost its extraordinary acuteness.

It is a well-known fact that the sense of smell is more acute in those persons who are deprived of any other sense, particularly sight. The odour of many bodies is not perceptible to most individuals, excepting under certain influences which increase its intensity. Such are friction, warmth, light moisture, electricity, solution in spirituous liquors, chemical changes, the morbid condition of anim al bodies, &c.

The sense of smell varies in different persons as much as that of taste. In some individuals it is unusually acute, as in a woman who could always foretel the approach of a storm by a sulphureous odour in the atmosphere. From various local or general morbid affections, the sense of smell may be increased or diminished in power.

The keen and discriminating power of the olfactory organ in various species of dogs is well known. They pursue their game, which flies before them at a considerable distance, without their true scent being at all interrupted by the various odoriferous bodies which must lie between them and their prey. Haller relates the story of a dog which followed his master who was more than a hundred miles distant, and then immediately discovered him amongst a crowd of people. There are many most extraordinary, yet well authenticated, facts in confirmation of the very acute olfactory powers of bloodhounds, and other animals. A traveller in Africa would have perished from thirst if a monkey, that accompanied him, had not led him to a spring at a considerable distance, and far beyond the sight. Neither the man nor the animal had ever been there before, and the fact is the more curious as pure water is nearly inodorous. The monkey might, indeed, be attracted by the smell of certain plants which grow near springs, and of which he possessed an instinctive knowledge.

Humboldt relates that, in Peru, large numbers of the Vultur are collected round a horse or cow slaughtered for the purpose, although there were previously none to be seen in the neighbourhood. It has also been said that the same bird has travelled from Asia to Thessaly, for the purpose of devouring the dead bodies on the field of battle.

The rapidity with which insects collect from a distance round sugar, honey, &c. is well known. Fish do not see the bait of the angler; they are attracted to it entirely by their acute smell. Every animal, and every organic and inorganic body, must have a peculiar odour, a specific emanation imparted to the surrounding atmosphere: otherwise the hound could not follow his master, and immediately distinguish the various things he had touched; nor could the ape be led to the spring at a considerable distance.

The acuteness of the sense of smell is in general in proportion to the size of the nostril. The odorific effluvia, or exhalation, which is given out by various bodies, extends to various distances. Bartholin declares that the approach to Spain may be ascertained at the distance of forty miles, by the smell of rosemary. The odorific particles which thus affect the surrounding atmosphere are so wonderfully minute, that, notwithstanding their immense numbers, the bodies from which they are emitted are not perceptibly diminished. Haller found that a bundle of papers, consisting of 400 dissertations, which had been perfumed with a single grain of musk, retained a powerful smell in every part after a lapse of forty years. In his Elem. Phys. v. 157, he makes a calculation of the very delicate division of the grain of perfume which had produced so lasting a scent through so many folds of paper. We are as yet incapable of estimating the weight* of such minute atoms of matter, or of explaining the essential distinctions between the exhaled particles which constitute such

* Prevost, of Genf, has invented an instrument called the Odoroscope, for the purpose of weighing the odorific exhalations from bodies.

vast varieties of odour. The division of the particles of light and caloric is still more subtle and incomprehensible than that of the odorific atoms, for they are enabled to penetrate glass and other bodies.

The odorific exhalation sent forth from the human body, proceeds either from the whole surface or from particular parts. It varies, under different conditions, both in quantity and quality. The trade, age, sex, and temperament of the individual,—climate, temperature, certain diseases, and passions of the mind, each produce variations in the smell of the effluvium which emanates from the body. At the period of menstruation or childbed, the odour emanating from the female is very peculiar. The food and particular manner of living have great influence upon the odour of the body. The French soldiers asserted that they could smell the Cossacks many hours after their departure from any place.

The effect produced upon the matter exhaled from the body by various mental emotions is very remarkable, particularly from rage, fear, or settled melancholy. By an attentive observation of changes produced by disease in the odour of the perspiration, the physician may frequently be enabled to form a more accurate opinion. A practitioner in Berlin, from having a very acute smell, is frequently enabled to determine the particular species of exanthematous diseases before the appearance of the eruption, entirely from the peculiar odour of the patients. Esquirol assures us, that, however attentive we may be to cleanliness in the persons of maniacs, there is always a specific odour arising from them. During many maladies, the acuteness of the sense of smell is wonderfully increased, and sometimes it is completely changed. A celebrated physician of Paris, who was confined to his bed, was constantly tormented by a metallic smell, which arose from a needle that had been lost in the bed-clothes. During inflammation of the brain, the sense of smell is particularly acute. Bailly, who was attacked with yellow fever, attended by much cerebral irritation, distinguished in the water which he drank the smell of the vegetables which grew upon the border of the river from whence the fluid was taken. The near approaches of death may often be known by the peculiar odour of the patient.

Almost every part of the human body has a particular smell, and the variable odour of the breath is well known. If the changes in this were more carefully observed, the diagnosis of the physician might be improved, particularly in febrile diseases. The author has related a case, in his *Anthopot. und Med. Erf.*, of a woman who could distinguish persons by the peculiarity of their breath. A very pungent and ammoniacal smell frequently proceeds from the feet, and, if it be suddenly checked, severe diseases are often produced. Very offensive exhalations occasionally proceed from the genitals. Dr. Vogel met with one instance in which, if the discharge on which this depended was suppressed by the application of cold, it took place vicariously from the axillæ. It sometimes ceased spontaneously. During its disappearance, the patient suffered severely from heartburn.

There are many cases on record of powerful passions and antipathies which have been excited by the peculiar odour exhaled from the bodies of particular individuals. There are many circumstances which confirm the opinion that there is combined with the insensible perspiration some electrical power which is distributed in the surrounding atmosphere. The author is acquainted with a lady from whose skin electric sparks are emitted whenever she changes her under garments.* It cannot be doubted that, by approaching a being intimately connected with such persons, other individuals will be more or less influenced. Certain antipathies which some persons have for cats and other animals, must be referred to an unusual subtilty of the sense of smell. Stanislaus, king of Poland, fainted whenever he entered a chamber in which there was a cat, although he could neither see nor hear it. A fellow student of the author's could also immediately tell if a cat was in the house, although he was in a different room.

---

### PATHOLOGY.

*Paraplegia.*—Mr. Earle has made some good observations on this subject of which we subjoin the most interesting.

In the sixth volume of the Transactions of the College of Physicians, published in the year 1820, there is a valuable paper by the late Dr. Baillie, entitled "Some Observations on Paraplegia in Adults." In this paper he states his belief that this form of paralytic affection had considerably increased in this country, although he could not assign any reason for it. He further states, that, "in adults, when the spine has not suffered from outward violence, paraplegia most commonly depends on a disease of the brain itself." This assertion is hardly warranted by any direct proof adduced by the author in the paper in question, as only one dissection is given where there was extensive effusion in the membranes and ventricles. It is true that, in some additional observations published in Mr. Wardrop's edition, he states, "Besides the account of the dissection published in my former paper, I have heard, from the most undoubted authorities, of one case where tumors were found in the brain, and of another where a large quantity of water was found in the ventricles of the brain, together with some in the theca vertebralis. To these (he says) I may add a third case, on the best authority, in which many of the arteries of the brain were found ossified, and a large quantity of water effused between the membranes and the brain."

From a long conversation which I had with the author in the beginning of the year 1822, I have strong grounds for presuming that these latter observations, which bear date April 1822, were partly in reference to some cases and dissections which I named to the Doctor, as he at that time distinctly avowed that his belief in the seat of these affections being in the brain did not rest on any additional anatomical facts, but was the result of his own reason. He was not aware, before that conversation, that I had published the dissections above alluded to, illustrative of this point, and appeared much pleased to find that I had confirmed the opinions he had advanced, by actual examination of the brain and spinal marrow. See p. 37.

It is not, then, without good reason that I venture to call in question the propriety of assuming it as an axiom, that "paraplegia in adults most commonly depends on disease of the brain." I admit that it is an occasional and not unfrequent cause; and, further, I admit that where such a train of cerebral symptoms exist as are described by Dr. Baillie, we should probably not err in referring the mischief to the head; yet I feel confident that the conclusion is too general, and would therefore warn practitioners against too

* Haller has related similar facts in his Elem. Physiol. t. v. p. 54;—and we have ourselves met with instances of it.—Ed.

hasty an adoption of it, as no doubt many instances occur in adults wholly dependent on diseases affecting the spinal marrow. The subject is one of much interest and often involved in obscurity, requiring the most minute attention to every particular, more especially when the more manifest symptoms of cerebral affection are absent. Having for several years directed my attention particularly to affections of the brain and spine, it may not be unacceptable to the members of this Society to present them with the result of my inquiries, illustrated with some original cases and dissections.

Although in many of these diseases it may not be possible to form a very correct opinion during life, yet there are many circumstances which, if closely attended to, will enable us to form a tolerably correct prognosis.

Paraplegia dependent on the existence of disease in the brain, generally occurs at the middle or more advanced period of life than is usual in diseases of the bodies of the vertebræ, or their intervening fibro-cartilages. Its progress is more rapid than the slow insidious approach of symptoms from the latter diseases; the affection is more general, occasioning more or less paralysis of the upper and lower extremities, and this will often take place in a very few days from the occurrence of the complaint. This disease happens much more frequently in men than women. The gait of persons suffering from cerebral affection is peculiar, and very different from that attendant on affections of the spine. It very nearly resembles the vacillating steps of a drunkard. Such paralytic persons are incapable of walking in a direct line; their limbs are loose, and thrown forward with an exertion of the whole body; there is a great consciousness of feebleness in walking, and the greatest difficulty in turning round. The appearance of the eyes often much resembles those of a drunkard, particularly when the patient is at all excited or anxious. The above analogy to the staggering steps of intoxication is readily understood, if we consider that it is the temporary disturbance of the brain, from the congestion of its blood-vessels, that deprives the drunkard of the power of directing his steps, and for the time induces a state bearing the closest resemblance to paraplegia.

Sensation is more impaired than in spinal affections, when it will often remain perfect after a total loss of the locomotive powers. This impaired sensation is often peculiar, imparting an idea of some foreign body, as a leather glove or stocking, being interposed. The patient appears to feel (if I may use the expression) through a false medium; the limbs are more wasted and flabby, without any spasmodic rigidity of the muscles, which so often occurs in affections of the spine. Although often accompanied with a torpid state of the bowels, aggravated no doubt by the impaired muscular power of the abdominal parietes, there has not, in any instance that I have witnessed, been any train of gastric symptoms similar to those which so constantly attend affections of the spine, especially of the dorsal region. In some instances there is the additional confirmation of an impaired state of some of the external senses, accompanied with vertigo, a sense of weight on the head, and a general disturbance of the cerebral functions. As disease advances, the power of the brain in transmitting its influence to the extremities becomes more and more circumscribed. Thus I have known a tubercular affection of the pia mater, in the first instance, cause a numbness and loss of feeling in the feet, which has gradually extended until all four extremities were completely paralysed, and the muscles concerned in respiration at length refusing their office, death ensued.

A very similar train of symptoms occurs in other diseases of the brain, particularly in cases of extensive membranous effusion from chronic inflammation, or effusion into the ventricles. In a case of fungus hæmatodes which I published in the third volume of the Society's Transactions, the same effect was produced; and I have since met with numerous cases of scrofulous tubercles in the substance of the brain, where the same phenomena were slowly induced, until suppuration has taken place, or the neighbouring membranes have become inflamed, when a fatal termination has rapidly ensued.

Whenever disease has proceeded so far as has been above described, one or more of the mental faculties has, in almost every instance, suffered; and no reasonable doubt can in such cases be entertained respecting the real seat of the disease. It is in slighter and more chronic cases that it is often difficult to form a correct opinion; yet to establish a correct diagnosis in such cases is of the utmost importance, both with respect to the prognosis we should form of the probable termination of the case, and with reference to the proper treatment to be adopted, that we may not subject the individuals to useless suffering from the application of caustic issues and setons to the spine, and the disappointment which would follow from their obvious inutility in such instances. (*Medico-Chirurgical Transactions.*)

---

### PRACTICAL MEDICINE.

*On the use of Belladonna as a Preventive of Scarlet Fever.*—The attention of the medical profession was some time ago directed to the employment of belladonna as a means of preventing the occurrence of scarlet fever, in those persons who might be exposed to the contagious influence of that disease. This practice was first adopted by HAHNEMANN, and has subsequently been still further investigated by many German physicians of eminence. The following are the results which have been derived from a considerable body of experience.

1. In a large majority of cases where the remedy was employed, the disease did not occur in persons exposed to its contagion.

2. The extension of the epidemic was prevented by the exhibition of the belladonna.

3. When the disease did occur after the use of that medicine, its severity was much diminished in most cases.

To procure the full effects of its preventive powers, it should be given in the following manner:—Three grains of the extract of belladonna are to be dissolved in one ounce of water, with a small quantity of spirit of wine. As many drops of this solution are to be given twice a day as the patient has years of age. To protect the constitution against the contagion effectually, the remedy must be continued for at least eight days. From peculiar idiosyncrasy, the belladonna occasionally fails in rendering the patient secure against scarlatina. It has appeared also that some epidemics are less under its control than others.—These observations are taken from HECKER'S *Litterarische Ann. der gesammten Heilkunde.*

From the mode in which the "German physicians" now recommend us to employ the belladonna as a preventive of scarlet fever, it is evident that the extremely minute doses at first recommended by Hahnemann have not been found effectual. He at first administered the 432000th part of a grain of the extract; but his subsequent experience induced him to believe that even this was too powerful a dose! In this country the peculiar doctrines of Hahnemann are but little known; but on the continent, and particularly in Germany, he has gained many converts. An interesting sketch of his general views of disease, and of his mode of administering remedies, is given in the *Journal des Progrès des Sciences Médicales*, tome i. 1827.

In addition to the above testimony of the German practitioners as to the preventive powers of belladonna against scarlatina, it may be satisfactory to add the results of Dr. Velsen's practice.* During an epidemic of scarlet fever in the town of Cleves, the belladonna was administered in six weeks to 247 patients. Out of this number, only thirteen contracted the disease:—Four children had taken the remedy irregularly; one child had taken it regularly for fourteen days; another had taken it eight days; and seven employed it only for forty-eight hours. Of these one, of a debilitated constitution, died. All the others had the disease very slightly, in comparison with those patients who had not taken the belladonna. The following striking proof is worthy of notice:—A man, the father of four children, was attacked with the prevailing epidemic after having visited a friend, who was confined to his bed with the same disease. His wife and children, the youngest three weeks old, the eldest four years, took the extract of belladonna regularly in the form and doses we have above mentioned. Although they continued to live with the patient, night and day, in a small and ill-ventilated apartment, they all escaped the disease.

---

*Propagation of Fever.*—Dr. Alison has made some important observations on this subject in the last Number of the Edinburgh Medical Journal. We subjoin the most interesting.

1. The very same houses and districts in which a succession of cases of fever is observed in one season, (chiefly in persons holding close intercourse with those already sick,) will remain, during a succession of other seasons, perfectly free from the disease, although inhabited by persons liable to it, although equally crowded, dirty, and ill ventilated, and although the disease be prevalent in other, even neighbouring parts of the town. This continual change of the seat of epidemic fever has always appeared to me nearly incompatible with the idea of its depending on a malaria. I have seen such successions of fever cases at one time or another, certainly in hundreds of places in the town, and I can say positively, not only that there is no one of these in which it prevails every year, but that there is no one from which it has not been absent for a succession of years within my own observation, even when prevailing in the vicinity.

The district in which, during the former epidemic, between 1817 and 1820 the greatest number of fever cases occurred to my observation, was a small part of the Cowgate and adjoining closes, 140 yards by 100, in which about 200 cases occurred in less than a twelvemonth. From many inquiries made there, I am certain that that district remained quite free from fever for several years together after that time; and, even during the present epidemic, I have not been able to hear of more than one house in that district in which fever has appeared, although I know two tenements just on its border in which it has been very prevalent, and caused several deaths.

* Journal Complement. Octobre 1827.

The hospitals in which fever patients are received in this town furnish striking examples of the same general fact. When Queensberry House was formerly occupied by fever patients, every resident clerk and every nurse in the house was successively affected with the disease; and, since it was re-opened in December last, the resident physician, two of the clerks, (who have not been resident, but have been several hours a day in the house,) the apothecary, several servants, and all the nurses except two, (in all above forty individuals, who had necessarily close intercourse with the sick there,) have had fever. If this be the effect of a malaria, it must be a very virulent and effective one, and it is reasonable to expect that some record of similar visitations in the former history of the building would be found. But Queensberry House has existed for about a century; it was long occupied as a private dwelling-house by the noble family of that name, afterwards it was occupied by a number of families, and afterwards as a soldiers' barrack; and yet no record can be found of its having been, during these changes, the seat of an epidemic fever. If a malaria has existed, therefore, in that house, it must, on both occasions, have sprung up exclusively at the times when fever patients were removed thither, and lasted only during their stay.

During the present epidemic, as well as in that of 1817-19, many of the clerks and nurses employed in the Royal Infirmary have taken fever. Since November last, six of the clerks employed in the clinical wards only, four of those employed in the ordinary wards, and twenty-five nurses or servants, have taken fever. All these nurses had necessarily frequent and close intercourse with the fever patients in the house, having been employed more or less constantly in the fever wards, excepting only four of the servants. Of these four, two had been employed in the laundry where the linen from the fever wards was washed; one was a porter employed at the gate, who would, of course, have communication with the fever patients at their entrance and dismissal, as well as with their relations coming to visit them; and one was a nurse employed in the servants' ward, but who was in the habit of visiting the fever wards.

Now, it is not only notorious that many years have often elapsed without any clerk, servant, or nurse, living in the Infirmary, or any of the students attending it, being seized with fever; but it is also certain that in this very season those of its inhabitants who have not had intercourse with fever patients have almost uniformly escaped the disease. Of the inhabitants of the ground floor of the house, (including the patients in the lock ward,) none but those already mentioned as having washed the linen from the fever wards, and the barber who shaved the heads of the fever patients, have taken the disease; yet in the case of a malaria it is the ground floor of a house that is generally found the most dangerous. No one of the nurses, whose duty has confined them to the medical or surgical wards, where no fever patients were admitted, has taken fever, with the single exception of the woman in the servants' ward above mentioned; and of the numerous patients in these ordinary wards, the only one who has taken fever, within my knowledge, during the present year, was a patient in the men's general clinical ward, who lay in the bed next the door that communicates with the clinical fever ward. If there be a malaria in this house, therefore, it would seem to restrict itself in point of space, as at Queensberry House in point of time, to the immediate vicinity of the fever patients.

2. While it appears that there is no part of the town subject to regular or even frequent visitations of fever, experience shows equally clearly that there is no part of it in which, when fever once occurs, a rapid succession of cases may not be observed, provided that frequent and close intercourse be kept up between the healthy and the sick. I have seen this myself under such circumstances in every possible variety of situation in Edinburgh and its vicinity, high and low, damp and dry, or even airy and confined, and often in situations widely different from those in which the malaria that produces intermittent fever is known to be chiefly generated. For example, when a person has fallen ill of fever, and lain some time in the upper story of one of the common stairs in the High-street, or adjacent closes, I have often seen a succession of other cases on the same flat, sometimes extending to the next below, while the inhabitants of the lower part of the same tenement, (sometimes of five or six stories,) have continued free from the disease.

In short, I have seen enough to convince me that, while there is no part of the town in which fever prevails regularly or very frequently, there is none in which it may not at times spread epidemically.

3. The succession of fever cases in limited districts takes place in some seasons much more rapidly and extensively than in others. But it is certainly a general fact, that it takes place, *cæteris paribus*, always more rapidly and extensively as the intercourse of the healthy with the sick is more close and intimate. Very close intercourse, even in well-aired rooms, is often followed by the effects in question, as appears from the number of nurses, and even of clerks, affected in the hospitals. Frequent and long-continued intercourse in close confined rooms is almost uniformly followed by this result: not in all those who hold such intercourse, but in a large proportion of them, and especially in young persons; and the more airy the rooms of the sick, and the less frequent the intercourse, the fewer instances of successive affection are observed. In the new town of Edinburgh, where almost all the houses are spacious and well aired, isolated cases of fever are common, and, when a succession of cases is observed, it rarely extends beyond a few individuals. But in the crowded and ill aired parts of the old town I can hardly recollect an instance, even during the years when the disease was least generally prevalent, of a patient in fever having lain at home during the whole disease without some other cases speedily following; and in many instances, when the disease has been more prevalent, the succession has extended to ten, twenty, or thirty, within a few yards of the residence of the first patient. All this corresponds exactly to the supposition of the disease being propagated by effluvia arising from the sick; but we know that the malaria arising from the earth, which engenders ague, is *much less* virulent in crowded than in thinly inhabited districts; and although it is certainly possible that there may be another kind of malaria generated within houses, or even in single rooms, and which may be increased by imperfect ventilation, yet it is hardly possible to conceive how the circumstances of crowding, or closeness of intercourse with the sick, should increase the efficacy of such a malaria, as they certainly do increase the tendency of continued fever to spread.

4. The efficacy of intercourse with the sick in producing the successions of fever cases, has seemed to me to be strikingly exemplified in several instances, in which fever has clearly appeared to be imported into particular districts, by persons whom I knew to have either had the disease themselves, or to have had close intercourse with the sick in other places. Two examples of this kind are I think worthy of being put on record.

Some years ago, at a time when there was no great number of fever cases in Edinburgh, I met with a case in the son of a shoemaker, who was lying in a room in which his father and two apprentices were at work. I could not prevail on the father to remove his son to the hospital, although I stated the danger of the apprentices being affected. Within two or three weeks after, I found that the two apprentices were lying ill of fever in their own houses,—one of them 200 yards, the other half a mile distant from the workshop, and widely distant from each other. These young men likewise lay at home during the fever, and each of their cases was speedily followed by a succession of others in the inhabitants of the rooms which they occupied, and of those immediately adjoining, who had never been at the workshop. In one of these houses seven, and in the other twelve, were thus affected. Now, on the supposition of the fever being contagious, all this was to be expected, and all corresponded to the predictions which were hazarded on that belief. But, on the supposition of such succession of fever cases depending on miasmata, there must have been at least two, more probably three, separate and accidentally concurring miasmata to explain the phenomena here observed: one at the workshop, and one at each of the houses of the apprentices; and there must have been this extraordinary coincidence, that at each of these last the malaria sprung up just at the time when a patient was lying there ill of fever, which he had apparently contracted elsewhere. Farther, the three houses in which these successions of fever cases were observed are in situations very different from one another; and all of them have been, to my knowledge, perfectly free from fever for years together, both before and since that time, notwithstanding that fever has been much more generally prevalent, and that they have been inhabited by successive families. What probability is there that three separate miasmata should have arisen in these three houses just at the time when their presence was required in each, to produce an effect which had been foretold as the consequence of another cause undeniably operating on all?

In the beginning of last winter, a family, consisting of a labourer, his wife, and four children, long out of employment, became affected with fever; the mother first. During her convalescence the father and two sons were sent to the Infirmary, and she and the others were turned out of their house when still very feeble, and found shelter with an acquaintance. When the father and sons left the hospital, the whole family again removed to a third house, considerably distant from the former, taking with them only some miserable dirty clothing. After many inquiries, I could not find that there had been previously any fever in any of the three places which they thus successively inhabited; but it is certain that many inhabitants of the same floor in which they first lived (and no others in that neighbourhood,) had fever immediately after them;—that, in the little court to which they next removed, thirty cases of fever occurred within a few weeks after they went thither, the inhabitants of the same room with them being the first affected;—and that from the third lodging-house which they occupied in the course of the winter, and within a fortnight after their arrival in it, four patients in fever were received into Queensberry House. Here again we must, on the hypothesis of a malaria, suppose three separate miasmata, and a wonderful accidental coincidence between the springing up of each and the application of the very cause which the other hypothesis assigns as the true explanation of the whole phenomena in question.

Lastly, the dependence of the successions of fever cases on the circumstance of intercourse with the sick, has appeared to me in many cases to be clearly indicated by the success of measures (especially the early removal of the sick,) taken to prevent the propagation of the disease, on the supposition of its proceeding from that cause, and which could not have a beneficial effect under any other supposition. This is a principle not susceptible of actual demonstration, but the probability of which increases so much on every repetition of the observation on which it is founded, as to amount ultimately to moral certainty. It is quite clear that, if the successions of cases in limited districts depended on a malaria in each, these successions should be equally apt to take place when the first patients were removed, as when they remained at home during their illness. But while it is, as I have already said, exceedingly rare to see a case of fever run its course in any of the crowded districts, without other cases speedily following in the immediate vicinity, nothing is more common than to see single cases not followed by any others, when they are removed to the hospital within a few days from their commencement. What was stated as the result of my experience in the New Town Dispensary during the former epidemic, is strictly applicable to all that I have seen in the present. "We should have little difficulty in pointing out above a hundred houses where a single case of fever has occurred, where the patient has been speedily removed and the place cleaned, and where there has been no recurrence. But we should hardly find five houses in all the closes of the old town in which a patient in fever has lain during the whole, or even during half of his disease, and in which other cases of the disease have not speedily shown themselves."

I have stated the results of my own observations on the cause of the propagation of the disease, without any reference to the statements of others; but it were easy to show—indeed it must have occurred to most readers,—that they correspond exactly with what has been observed by those authors whose writings on the subject of contagion are most esteemed in this country. Taking all the facts above stated together, and comparing them with the concurring testimony of many other observers, I must profess myself to be not only satisfied with the evidence on this point, but also at a loss to understand what better evidence can possibly be expected. At all events, it is plain that the evidence rests entirely on *comparative observations*, and is very different from that which is furnished merely by the *simple observation* of a number of fever cases occurring simultaneously or successively in a limited district; and that it cannot be refuted merely by pointing out the fallacy attending conclusions drawn from such simple observations only.

No doubt it is difficult to understand why, of a given number who have intercourse with the sick, some only should be affected, and others should escape; but the same difficulty attaches itself to almost every other instance in which a disease is referred to its exciting cause, there being no cause of disease which acts with perfect certainty and uniformity. And at all events the same difficulty attaches itself to the explanation of the diffusion of fever by the supposition of a malaria. To start this difficulty, is in reality to express a doubt whether the fact of the successive affections of persons living near one another is one so generally observed as to demand an explanation at all; whereas it is allowed, I believe, by all inquirers, that this fact, though not a constant and invariable one, is yet one so generally observed as to demand some explanation; and the only question is, what explanation is the most consistent with the phenomena?

It should not be overlooked, in any discussion regarding the contagious nature of fever, that in those seasons when it prevails most epidemically, and very remarkably in the present, it is very frequently attended by an eruption of the skin, closely resembling what is seen in some of the contagious exanthemata, particularly the measles. A majority of the cases treated by me in the hospitals during the present year had more or less of this eruption. It was most distinct and numerous in severe typhoid cases, but not confined to cases of any peculiar type. It was very irregular in its time of appearance and disappearance; seldom appearing, however, before the fourth or fifth day, sometimes not till the tenth or twelfth; in which last case it was attended, I think uniformly, by an aggravation of the febrile symptoms, and was of course a very unfavorable sign. It very seldom assumed the appearance of true petechiæ. Such cases of spotted fever may be said to form the link that connects the order of fevers with that of contagious exanthemata.

---

*Employment of Iodine in the Treatment of Cynanche Parotidœa.*—Dr. Neumann, of Neustadtel, in Silesia, has employed the hydriodate of potass with great success as an external application in cynanche parotidœa, which prevailed epidemically in Neustadtel in June 1823. Among the poorer orders who were treated in the ordinary way, the disease was very tedious, and generally ended in suppuration. Among the better ranks, the treatment consisted in the exhibition of an emetic, and the application over the tumor of a plaster composed of eight parts of mercurial ointment and one part of the hydriodate of potass; and the ordinary effect was a radical cure within three or four days. Dr. Neumann adds, that he never observed in the cases so treated any metastasis to other organs; and he is disposed to impute this to the formation of an erythematic eruption, which always appeared on the first or second day, and remained from eight to twelve days.—The author has annexed a very instructive case illustrating the deleterious effects of this drug when given in too great quantity. It was given to cure enlargement of the cervical glands, which it accomplished very effectually; but the patient was at the same time attacked with violent palpitation in the chest and abdomen, which was relieved only by the horizontal posture, then with great weakness and tendency to faint, and subsequently with emaciation and general dropsy. The palpitation was so violent and constant as at first to appear connected with organic derangement; but the relief obtained from the horizontal posture, the absence of cough and dyspnœa before the dropsy commenced, and the regularity of the pulse, led Dr. Neumann to conceive that the disorder of the heart was merely functional. Accordingly, although the patient had been ill for a twelvemonth, the irregularity of the heart's action was soon subdued by the employment of digitalis and cherry-laurel water: and the tendency to faint and anxiety at the same time disappeared. The dropsy, however, continued much longer; but at length it slowly receded under the use of the Arnica combined with soap. (Rust's *Magazin für die gesammte Heilkunde.*)

---

*Bite of the Viper.*—M. Jacopo Sacchi, of Barzio in Valsasina, having had occasion to take charge of some cases in which injury has been inflicted by the bite of a viper (Coluber Berus), transferred his observations upon them into the hands of Professor PALETTA. From these it appears that ammonia, recommended by Dr. MANGILI in 1813, although an exeellent remedy in many cases, is by no means sufficient in all, but must occasionally be seconded by every possible means. Although sometimes nature alone has power sufficient to overcome the bite of a viper, yet at other times the injury is so great and sudden, as to resemble the effects of hydrocyanic acid. In these cases he recommends that the patient should be put into a hot bed covered with woollen clothes, and the most powerful sudorifics, with some tonics, administered internally. Friction should be applied all over the body, and at the same time the wounds are to be enlarged, cupping-glasses applied, and tow dipped in ammonia applied to the spot. (*Journal of Science.*)

### SURGERY.

***Dislocations of the Vertebræ.***—The following extract is from a paper by Mr. LAWRENCE, in the *Medico-Chirurgical Transactions.*

Complete dislocations of the vertebræ without fracture, whether produced by accident or disease, are so uncommon, if we except the first and second bones of the neck, that the possibility of the occurrence has been doubted, and even denied, by some of the highest authorities. In the Traité des Maladies Chirurgicales, which contains the valuable results of his long experience, Baron Boyer points out the circumstances which prevent the displacement of the bodies of the vertebræ, and, without expressly denying it, appears clearly to disbelieve its possibility.* He asks the question, whether the bodies of the vertebræ can be luxated?—alludes to the kind of accidents under which such displacement has been considered probable;—and then, having shown the powerful obstacles to it, concludes by observing, that, if we examine the cases in which dislocation has been supposed to have occurred, we shall find invariably that the posterior laminæ of the vertebræ have been broken; that in many instances they have been comminuted; and further that, when the body of a vertebra has undergone any displacement, the laceration of the fibro-cartilage has been almost always accompanied by detachment of some portion of the bony substance.

Delpech represents it as a point proved by observation, that the bodies of the vertebræ cannot be dislocated.†

Sir A. Cooper‡ says, that "if luxation of the spine ever does happen, it is an injury which is extremely rare, as in the numerous instances which I have seen of violence done to the spine, I have never witnessed a separation of one vertebra from another through the intervertebral substance, without fracture of the articular processes; or, if those processes remain unbroken, without a fracture through the bodies of the vertebræ."

Other surgeons, however, have affirmed that the vertebræ may be luxated. After pointing out, in his Treatise on Diseases of the Joints, the circumstances which render this occurrence very improbable, Rust affirms that even the lumbar and dorsal vertebræ may be dislocated, and refers to instances recorded by various writers.§ He mentions the following as a case of dislo-

* Tome iv. chap. iv. article iv.

† Précis Elémentaire des Maladies reputées Chirurgicales, tome iii. p. 42.

‡ Treatise on Dislocations and on Fractures of the Joints. First edition, p. 539.

§ Arthro-kakologie, p. 71.

cation in the cervical region of the spine, successfully replaced by himself:—"The injury was produced by a severe fall on the head. The neck was completely bent to the right side, the upper extremities already paralysed, and repeated attacks of convulsions and hiccough came on. Replacement was immediately attempted, and succeeded. I made the patient sit on the ground, and had the head drawn straight upwards by a strong assistant. The patient got well under the employment of cold locally.*

Mr. Bell mentions a case of complete dislocation between the last dorsal and first lumbar vertebra, with entire division of the spinal cord. A small portion of bone was broken off. He gives two views of the parts.†

The greater mobility of the individual bones, the comparative smallness of their bodies, and the obliquity of the articular processes, point out the cervical vertebræ as those most likely to be luxated: at the same time the form of the neck, and its connexion with the head, are favorable to the application of such violence as may cause luxation. Hence not only does dislocation of the atlas occasionally occur, but we have also instances of luxated articular processes in the case of the five inferior cervical vertebræ. Baron Boyer even considers that this may happen without external violence, and that the inferior articular process of a cervical vertebra may be carried in front of the superior articular process of the vertebra below it, by a sudden and forcible rotation of the head and neck towards the opposite side. He says that "Desault mentioned in his Lectures the case of an advocate, who met with this kind of dislocation by turning his head suddenly round to see who was coming in at a door situated behind his seat. Chopart also showed us a young man, twenty-four years old, in whom a similar accident had occurred, in consequence of an extreme rotation of the head, leaving the head permanently inclined upon the left shoulder."‡

Baron Boyer mentions another instance, which Desault used to relate in his Lectures, of a child, eight or nine years old, who, in turning heels over head on a bed, luxated the right lower articular process of a cervical vertebra. The head was turned towards the left shoulder, and so firmly fixed that it could not be brought into its natural position, even by the employment of considerable force. When the proposal of attempting reduction was made, Desault decidedly discountenanced it.§ Another case of a child terminated fatally, under the employment of efforts at reduction; and, when the body was examined, one of the inferior articular processes of a cervical vertebra was found dislocated forwards.‖ It does not appear that Baron Boyer had ever ascertained the existence of this dislocation by actual examination, and it is even doubtful whether he had ever seen a case at all. Further, he admits that no example can be adduced of both articular processes being dislocated at the same time.

Sir A. Cooper¶ says that he has never seen a dislocation of the cervical vertebræ: he does not deny the possibility of the occurrence, but seems hardly to believe it.

In the anatomical museum at St. Bartholomew's Hospital there are specimens of luxated cervical vertebræ. In one of these the right inferior articular

* Medicinisch-chirurgische Zeitung, 1813, vol. iii. p. 127.

† On Injuries to the Spine and Thigh-bone, p. 25. Plate ii. fig. 2 and 3.

‡ P. 114. § P. 117. ‖ P. 118.

¶ On Dislocations, p. 539.

process of the fifth vertebra is dislocated forwards. The portion of the vertebral column above the seat of the injury is twisted to the left, and the body of the fifth, having been partially displaced, projects beyond that of the sixth vertebra. This displacement could not have been effected without laceration, or considerable injury of the fibro-cartilage. The upper and anterior part of the body, both of the sixth and seventh vertebræ, has been slightly fractured on the left side, without detachment of the broken portion. It appears as if a small piece of the edge had been broken off by the twisting of the spine to the left. As the specimen, which is dried and inclosed in a bottle, has not been completely cleaned of the soft parts, the exact nature and extent of the fracture cannot be ascertained; nor can it be pronounced with perfect certainty that there is no fracture of the processes. For the same reason, we cannot now make out the precise degree and kind of injury sustained by the fibro-cartilage.

In another, the inferior articular processes of the fifth cervical vertebra are partially separated from those of the sixth, having been drawn upwards; but they have not been thrown forwards. The bodies of the same vertebræ are partially separated behind, but they preserve their natural level and relative position in front. The connexions of the two vertebræ must have been torn by a force applied behind, bending the neck powerfully forwards, and the bones must have subsequently resumed their natural position, so that there would have been no pressure on the spinal marrow. This kind of injury has been called, by Mr. Bell, Diastasis, or sub-luxation.§

A third specimen exhibits a dislocation of the sixth upon the seventh cervical vertebra. The inferior articular processes of the sixth are completely displaced forwards, and its body projects over that of the seventh. The spinous processes have been sawn out, so that it is uncertain whether they were injured or no. This may be deemed a case of complete dislocation; but there is an appearance of fracture near the basis of the articular process, the exact nature of which cannot be ascertained in the present state of the specimen. Mr. Stanley considers that this fissure was made by the saw in removing the spinous processes.

---

*Cure of Nasal Polypi.*—Dr. Primus, of Babenhausen, asserts that the saffronised tincture of opium (of the Prussian Pharmacopœia) possesses the property of gradually destroying nasal polypi, when applied to them. Certain cures, which have been thus effected, have already been published, and a striking case occurred in January 1826. A man, forty-six years of age, had one in each nostril. The tincture was applied several times a day to the bases of the polypi, by means of a small hair-brush or lint roll. In eight days the tumors had assumed a paler appearance, and lost a little in volume; a serous secretion from the nose, which had existed for a long time, was diminished, and the pituitary membrane had acquired a more lively tint, as if in a sub-inflammatory state. The application was continued, the tumors continued to decrease, and at the end of three weeks had entirely disappeared. (*Mediz. Chirurg. Zeitung.*)

---

*Cold Water as a Remedy in severe Salivation.*—We have received a letter from Dr. Andrew Haynes, of Vicksburgh, Warren County, Mississippi, recommending the application of cold water to arrest severe salivation. He writes that he has witnessed "the most decided and prompt abatement of the swelling and inflammatory symptoms in several cases, from pouring a gallon of cold water gently on the head and neck." "In cases," says he, "where gangrene was to be apprehended from the severity of the inflammation, I have prescribed the cold water to be repeated every three hours, or oftener, with decided advantage."

We have no experience with the remedy in such cases: it is a well-known fact, however, that cold and moisture are powerful agents in developing the mercurial action, and it will be remarkable if further experience should confirm its power to arrest its progress. The late Professor Rush, in the yellow fever that prevailed in this city, in many cases where mercury failed to affect the system, and in which it was important that this should be speedily affected, applied to the neck of the patient cloths wet with cold water, and generally with the desired result: indeed, sometimes the most profuse salivation was produced in an exceeding short period. (*Philadelphia Journal.*)

---

Barton's *new Operation for Anchylosis.*—Dr. Barton was kind enough to bring his patient to us today (July 20th), that we might examine him. The motions of the new joint are more extensive than they were when we last saw him. He flexes his thigh on the pelvis more than he could, perhaps to an angle of about 120°: of course, when sitting, he is still obliged to bend his spine. The motions of the joint appear to give him no pain; he walks with a short stick, and without limping much. He walked several times across the room without the aid even of his stick, but limped a good deal in doing so. He went up and down a flight of steps, with the aid of his short stick only, with great ease. His two limbs are nearly of the same length, but, when standing, the side of the pelvis over the affected limb is somewhat elevated. When his legs are crossed, and his feet close to one another, he stands firm, and appears to bear an equal portion of his weight on each limb; but when his feet are together, with his legs parallel, he bears his weight principally on the sound (left) limb, and the pelvis of the right side appears elevated.

He informed us that a few days since he walked from his residence to the hospital and back again, a distance of three miles. Occasionally, he amuses himself with his gun, and Dr. Barton was shown, on a recent visit to him, a number of birds that he had shot. After using much exertion, he (Coyle) says that he suffers from general fatigue, but does not experience pain in the new joint. The operation must be considered as eminently successful, and is highly creditable to the boldness, ingenuity, and skill of the operator. (*Ibid.*)

---

*Syphilis and Phagedena.*—The following distinctions between these are made by Mr. Welbank:—

Syphilis in general is first observed at a more remote period from sexual intercourse than other venereal diseases. It commences in some point of inflammation, which is soon characterised by abrupt induration. It proceeds, sooner or later, to indolent and uniform ulceration of the surface, between which and the edge there is no undermining groove of separation. The inguinal glands are little, and often not at all, disturbed by the absorption of the virus, even when secondary diseases have already manifested themselves.

The secondary diseases of syphilis arise in the series noted by Mr. Hunter, attacking first the skin and throat, and then deeper-seated parts. They are ushered in by a soft and much accelerated pulse, without any other obvious febrile symptom, and by severe nocturnal pains. They are indolent, and slow of progress. They consist of an eruption of firm and slightly elevated spots, from which pellicles or scales are from the commencement successively detached. These spots are thick about the scalp, chin, forehead, and upper and inner part of the thighs. In situations where there is hair, they frequently form slightly elevated crusts of a pale colour. When they appear on the palms of the hand or soles of the fees, they are characterised by a thick honeycomb desquamation of the dense cuticle. They are more disposed to superficial ulceration, when confluent or situated between opposed and secreting surfaces, as the angles of the mouth, scrotum and thigh, the nates, or between the toes. The ulceration of the tonsil is attended with little pain at first, and excavates the part affected deeply and often in a triangular form, as if the tonsil were split. This ulceration slowly acquires, especially on the supervention of simple excitement, a smooth buffy surface. Syphilitic nodes are a late symptom, and appear in the form of a painful but chronic enlargement of the bone affected. Syphilis is a rare disease, and is in every form directly superseded *pro tempore* by the excitement of mercurial irritation. It is long intractably stationary, or even progressive in most cases, unless mercury or sarsaparilla be employed.

Phagedena may arise spontaneously in cachectic habits, either in its primary or secondary forms. Sometimes it is first noticed many months after sexual intercourse, sometimes on the day following; it exhibits in general a rapid inflammatory ulceration, succeeding to pustule, extensive excoriation or fissure of surface. Frequently there is puriform discharge from the urethra. This is often recorded, under the name of Gonorrhœa, as the cause of secondary diseases. It is, however, in many cases only a concomitant mark of a certain cachectic constitution. It sometimes first appears contemporaneously with the supposed secondary diseases, sometimes three or four weeks after primary ulceration of the genitals. Sometimes it recurs many months after supposed cure, in conjunction with eruptive diseases. It differs both in progress and cure from simple gonorrhœa, and may have appeared ten or twelve times in one patient, from constitutional liability. Primary phagedenic ulcerations are various and multiform, and do not always exhibit a continued disposition to extend themselves. They in general spread at their edges, in parts or the whole of their circumference, and are characterised by considerable tumefaction of cellular tissue, and frequently phymosis. Natural processes of reparation in milder forms of the disease quickly follow those of destruction. The secretions in phagedenic ulceration are, for the most part, sanious or purulent, and ordinarily profuse. The inguinal and other absorbent glands become subject in many cases to rapid enlargement and suppuration. These affections of the absorbents often arise when the exciting sores are healing or have healed.

The constitutional diseases of phagedena are frequently coexistent, and even simultaneous, in their origin with those termed primary. They do not attack different structures in any regular series, and even nodes are contemporaneous with cutaneous and other diseases, or even precede them. Some of them often exist in an active state, with little disturbance of the circulation, and are generally characterised by rapid or sudden local inflammations. The affections of the skin consist of distinct purplish or brownish spots, with or without elevation, of a thick, papular, vesicular, and pustular eruption on the trunk and extremities, the three forms being generally conjoined. These eruptions are not found on the palms of the hands and soles of the feet, and exhibit an appearance of desquamation on their decline only. Sometimes there are distinct superficial tubercles slightly vesicating, or large phlyzaceous pustules forming crusts. When the phagedenic virus attacks the cellular tissue, it causes deep red and much elevated tubercles, of various sizes and furunculous character, the larger of which often exhibit a considerable extent of phlegmonous inflammation. A superficial and rapid ulceration of phagedenic character exposes the sloughy cellular tissue, which leaves a deep cavity on its separation. Frequently may be noticed a combined affection of the skin and cellular tissue, attended with superficial ulceration, which extends at its edge, while it heals in its centre, forming an islet or peninsula of skin. These sores, which are frequently covered with prominent crusts of rupia, resemble in shape the base of the horse's foot, and leave a white depressed cicatrix, circular or oval, and very characteristic of phagedenic ulceration. Sometimes large patches of integument become red and indurated, and subsequently ulcerate superficially into many circular, oval, and crescentic sores. These are especially frequent about the carpus and tarsus, and round the elbow, ankle, and wrist joints, and on the thigh and calf of the leg. The phagedenic ulcerations of the throat and fauces suddenly occupy a considerable space, exhibit often a shreddy irregular surface, and a bright red and everted edge. Sometimes the phagedenic process is limited to the mucous membrane, which is grooved out by the advancing edge, while processes of reparation are visible in the parts first affected. Suddenly suppurating inflammation occurs, even at early periods, about and beneath the periosteum of various bones, particularly of the cranium, nose, and tibiæ, and about the carpus and tarsus; this symptom being more generally observed where mercury is indiscreetly employed. Intense and agonising pain attacks the external parts of the head, in many cases. With the eruptions of the skin is often joined iritis. Inflammation and ulceration of the larynx, enlargement of the testicles, effusion into the joints, especially of the knee, and phagedenic ulceration about the lacrymal sac and eyelids, may be added to this long catalogue of disease; and sometimes there exists extensive herpetic ulceration of the skin of the face and forehead. This affection often destroys in its progress the alæ and septum of the nose.

Mercury administered so as to affect the circulating system is superfluous in the milder symptoms, and exasperates generally the more severe. Even when apparently yielding to an increased action of mercury, the maladies suddenly relapse, and are often protracted in their duration. The disposition of many of the milder forms of this disease to processes of natural cure and reparation, is a frequent source of error in estimating the utility of mercury, and even the more severe forms are in some instances, for a time, superseded by pushing, as it is termed, a mercurial course. Each relapse, however, of disease, especially in its more formidable shapes, is aggravated in severity proportionably to the disturbance of the constitution unnecessarily induced by such injurious treatment, till at length an irremediable state of disease is established. Iritis is the only affection in which the risk of irreparable injury by delaying the use of mercury, counterbalances the almost certain evil of administering it so as materially to disturb the circulating system. (*Medico-Chirurgical Transactions.*)

*Amputation of the Thigh at the Hip-joint*, by Mr. Orton.—Having made the necessary arrangements, with Mr. Oldknow on my right hand, and Mr. Attenborough on my left, and standing on the right side of the patient, an assistant forcibly brought down the thigh, which was previously drawn up close to the abdomen, when I carefully cut down to, and passed a strong ligature round, the femoral artery, about two inches below Poupart's ligament. That being done, I plunged a long double-edged catlin directly through the upper part of the thigh, in a direction downwards as the patient lay on his back, the point entering close by the neck of the os femoris, and coming out near the tuberosity of the ischium, and, cutting downwards about two-thirds of the whole length of the thigh, keeping the knife close to the bone, I brought it out in a somewhat slanting direction; thus forming, by one incision of the muscles on the inside of the thigh, a large fleshy flap, which was immediately grasped by Mr. C. Attenborough. Then, changing the knife for a scalpel, I laid open the joint to as great an extent as I could, and the assistants forcibly abducting the thigh, so as to thrust the head of the bone forwards, I succeeded in dividing the ligamentum rotundum without difficulty. The capsular ligament being, however, as yet only divided on the inside, I resumed the long knife, intending to have passed it between the head of the bone and the acetabulum, and by holding the point directly downwards as when the first thrust was made, to have cut through the muscles and integuments from within outwards. But not being able to get the knife through the joint in that manner, I was obliged instantly to change my purpose, and carrying the handle of the knife downwards and outwards, cut through the integuments and muscles all round, from the acetabulum in front, directly above the trochanter major, down to the tuberosity of the ischium below. I had then only to divide the capsular ligament on the outside of the joint, together with the tendons inserted into the hollow behind the trochanter, and the limb was severed from the body.

Mr. Attenborough immediately placed his other hand over the middle of the wound, to prevent hemorrhage there; whilst Mr. Oldknow and myself began to secure the vessels with ligatures as quickly as possible, beginning with those on the outside of the joint, proceeding then to those about the acetabulum, which had been compressed by Mr. Attenborough's left hand, and lastly to those of the large flap, which had been held in his right. But little blood was lost, and one-third of that came from the femoral vein. About a dozen ligatures were found necessary.

On examining the wound, there were found two large sinuses extending into the muscles covering the dorsum ilii, where matter had lodged; but they were not cut out, nor was any thing done to them; nor was the cartilage, &c. about the acetabulum pared off. The vessels being secured by ligatures, and the wound simply cleansed, the large flap was brought over the wound, which it covered exceedingly well, and was secured, first by about half a dozen ligatures, and then by straps of adhesive plaster; after which, a double-headed roller was applied, and the poor fellow put to bed as well as could have been expected.

A state of syncope occurred early in the operation; but one of the gentlemen, who kindly took charge of him while we were engaged with the limb, gave him a little wine occasionally, as he seemed to require it, and he passed through it very fairly. (*Ibid.*)

The patient recovered after a very tedious convalescence.

### MIDWIFERY.

***Parturition.***—Upon a few occasions, Dr. MERRIMAN has known the period of delivery, dated from the last appearance of the catamenia, has exceeded forty-four weeks, or 308 days; and we subjoin a short detail of these anomalous cases.

1. Mrs. I. had in ten pregnancies borne eleven children: she had on all these occasions become pregnant almost immediately after the monthly intermission. She was regular in March 1813, and had no appearance after the 7th instant. She was led to believe that she conceived on the 8th, and made preparations for her confinement in the early part of the following December. Her labour did not take place till the 11th of January, 1814, making 309 days, not including the day of supposed conception, since the disappearance of the catamenia.

The child, a boy, when born, was larger than most, if not than any, of her former children, and her labour was of several hours' longer duration. She was once pregnant afterwards, but this pregnancy presented nothing unusual.

I have always been inclined to think that in this case gestation was protracted beyond the usual term of forty weeks. The lady was a woman of great modesty and good sense, and free from every sort of affectation. She could have had no motive for fixing upon the 8th of March as the time of conception, but a firm conviction that she was right in her calculation; and neither the time of quickening nor any other circumstance induced her to suspect that she had formed an erroneous opinion.

It has indeed been assumed respecting this case, that the lady did not conceive on the 8th of March, but much later in the interval of freedom from menstruation: namely, only just before the next recurrence of the period. Ought this assumption to be admitted as decisive against the opinion of the lady herself? She had formed her opinion after the experience of ten pregnancies, in all of which she calculated from the same premises, and her reckoning had proved correct; and in a subsequent pregnancy, calculating in the same way, she again formed a correct opinion. The assumption rests altogether upon the hypothesis that in this one particular the usual course of nature cannot be deviated from, though we have evidence *ad infinitum* that the course of nature in every other point relative to conception, gestation, and parturition, is continually interrupted and deranged. But let us grant what is assumed, that the conception did not take place till "the day preceding the next menstruation," will this clear up the difficulty? The catamenia had ceased on the 7th of March: according to the laws of nature, as contended for by those who make this assumption, the catamenia, unless arrested by conception, ought to have returned at the end of twenty-eight days, consequently on the 4th of April, but they did not return on that day; and therefore, according to this hypothesis, the conception must have been complete on the 3d of April, which gives, to the 11th of January following, 283 days; and demonstrates that the longest period of pregnancy which the objectors allow to be possible was exceeded by three days.

2. Mrs. N. had lain-in of one child, and the number of days which elapsed on that occasion between the catamenial intermission and the labour was 303. Mrs. N. was unwell in November 1822; she recovered on the 15th, and had not the slightest appearance afterwards. Her labour took place on the 5th of October, 323 days from the day of intermission.

It should be remarked, that Mrs. N. is of a very nervous temperament, and has always been irregular in her periods of menstruation. The child was large, but not larger than might have been expected from a mother who is herself above the usual size of females. This case, therefore, though exhibiting so considerable a deviation from the usual course of pregnancy, cannot be adduced as satisfactory evidence of protracted gestation.

3. Mrs. B., upwards of forty years of age, who had not borne a child for more than nine years, was unwell in March 1823. As there was no appearance of the catamenia in April, nor the following months, she comforted herself with the hope that the critical change in her life had been happily effected. After some considerable time, however, she began to enlarge in size, and, fearing that some disease was forming, she consulted the late Mr. Chevalier, who probably supposed that her complaint was ovarian.

The enlargement continuing to increase, she was recommended to procure the advice of an accoucheur, and in consequence applied to me. On hearing the history of the case, and being positively assured that there had been no appearance of the catamenia for more than twelve months, there was no reason to suspect pregnancy, and I concluded, therefore, that the enlargement was occasioned by an ovarian tumor. When, however, I had other opportunities of seeing Mrs. B., and was permitted to make an examination per vaginam, it became evident that pregnancy was considerably advanced, and in nine or ten weeks afterwards (viz. September 27, 1824,) she was delivered of a very stout healthy boy.

---

### CHEMISTRY.

*On the Iron contained in the Blood.*—Mr. Englehart has shown that when an aqueous solution of the colouring matter of the blood is treated with chlorine, it is decomposed; a flocculent substance is deposited, which is insoluble in water, and which, when separated by the filter, yields all the iron of the colouring matter, by treating it with the commonly employed re-agents.

Mons. H. Rose, has repeated and verified the experiments of Englehart. He found, however, that the iron precipitated by ammonia contains a small quantity of phosphate of lime and subphosphate of iron, and also that if excess of ammonia be added without separating the flocculent matter, it is re-dissolved; the liquid becomes of a deep brownish-red colour, and the iron does not precipitate. After a long time it is indeed true that flocculent matter is deposited, but it contains scarcely any iron, almost the whole of it remaining in the ammoniacal solution. It appears, therefore, that the red colouring matter and the flocculent matter derived from its decomposition by chlorine, possess the property of hindering the precipitation of the iron by the alkalies; indeed, a considerable quantity of solution of iron may be added to the colouring matter, without its being precipitated by the alkalies. If the colouring matter be destroyed by chlorine, and the insoluble matter be separated, all the iron may be precipitated by re-agents; but this is not the case if the insoluble matter be left in it. There is however a limit to these facts; for when the iron is in too great proportion to the colouring matter, the iron is then partly precipitated.

M. Rose found that when the serum of human blood, or of the ox or sheep, was mixed with a considerable quantity of solution of iron, it presented the action of the usual re-agents upon the metal. Ammonia and the other alkalies, although added in great excess, produced no precipitate: they even re-dissolved that occasioned by the addition of albumen to a solution of peroxide of iron; and neither hydrosulphuret of ammonia, nor tincture of galls, occasions any precipitation in the solution.

In general, peroxide of iron and other oxides are not precipitable by alkalies, when an organic substance soluble in water is added to the solutions, provided it be of such a nature as to be entirely decomposable at a high temperature. On the contrary, when an organic substance soluble in water is totally or mostly volatilized without decomposition, it does not prevent the iron from being precipitated by the alkalies. The first effect is produced in a hot solution of gelatine or starch, in gum arabic, linseed, mucilage, sugar, sugar of starch and of diabetes; the pectic, kinic, malic, citric, and tartaric acids; in fact, this last acid has long been known to produce this effect. The contrary property was found in the following acids; viz. the oxalic, acetic, formic, pyro-tartaric, pyro-citric, pyro-mucic, succinic, benzoic, &c., and by alcohol and sulphuric ether.—*Annales de Chimie et de Physique.*

---

*New Alkali in Hemlock.*—Professor Ficinus, of Dresden, has discovered a new alkali in the Æthusa Cynapium (Linn.) to which he has given the name of Cynopia. It is crystallizable, and soluble in water and alcohol, but not in ether. The crystals are in the form of a rhombic prism, which is also that of the crystals of the sulphate. (Hensman's *Repertoire.*)

---

### MISCELLANEOUS.

*Paper to resist Humidity.*—This process, which is due to M. Engle, consists in plunging unsized paper once or twice into a clear solution of mastic in oil of turpentine, and drying it by a gentle heat. The paper, without becoming transparent, has all the properties of writing paper, and may be used for the same purposes. It is especially recommended for passports, workmen's books, legal papers, &c. When preserved for years, it is free from injury, either by humidity, mice, or insects. It is further added, that a solution of caoutchouc will produce even a still better effect. (*Kunst und Gewerbe-blatte.*)

---

*Indelible Writing Ink.*—Let a saturated solution of indigo and madder in boiling water be made in such proportion as to give a purple tint; add to it from one-sixth to one-eighth of its weight of sulphuric acid, according to the thickness and strength of the paper to be used: this makes an ink which flows pretty freely from the pen, and when writing which has been executed with it is exposed to a considerable, but gradual, heat from the fire, it becomes completely black, the letters being burnt in and charred by the action of the sulphuric acid. If the acid has not been used in sufficient quantity to destroy the texture of the paper, and reduce it to the state of tinder, the colour may be discharged by the oxymuriatic and oxalic acids, and their compounds, though not without great difficulty. When the full proportion of acid has been employed, a little crumbling and rubbing of the paper reduces the carbonaceous matter of the letters to powder; but, by putting a black ground behind them, they may be preserved, and thus a species of indelible writing ink is procured, (for the letters are, in a manner, stamped out of the paper,) which might be useful for some purposes, perhaps for the signature of bank-notes. (*Journal of Science.*)

---

*Proceedings against Empirics in Edward the VI.'s Reign.*—In this king's reign, several practisers of physic were examined by the College, and found so unfit for the practice of that art, that they were rejected; others were punished according to public statutes, and others fined.

In the fourth year of the king's reign, in the month of September, one Grig, a poulterer, of Surrey, taken among the people for a prophet, in curing of divers diseases by words and prayers, and saying he would take no money, &c. was, by command of the Earl of Warwick, and others of the council, set on a scaffold in the town of Croydon, in Surrey, with a paper on his breast, wherein was written his deceitful and hypocritical dealings; and after that, on the 8th of September, set on a pillory in Southwark, being then our Lady Fair there kept; and the mayor of London, with his brethren the aldermen, riding through the fair, the said Grig asked them and all the citizens forgiveness.

"Of the like counterfeit physician, saith Stow, have I noted (in the Summary of my Chronicles, anno 1382,) to be set on horse-back, his face to the horse-tail, the same tail in his hand as a bridle, a collar of jordans about his neck, a whetstone on his breast, and so led through the city of London, with ringing of basons, and banished." (GOODALL's *History of the College of Physicians.*)

---

*Medical Report during Lady Jane Seymour's Illness.*—She was married to Henry VIII. the day after Ann Boleyn's execution. Seventeen months afterwards she fell in labour, and was delivered of a living son on the 12th of Oct. 1537. She was alive on the 17th. The Herald's Office dates her death on the 24th Oct. The following is the report of her six physicians:—"These shall be to advertise your lordship of the queen's estate. Yesterday afternoon she had a natural *laxe*, by reason whereof she began somewhat to lighten, and, as it appeared, to amend, and so continued till towards night. All this night she hath been very sick, and doth rather appaire than amend. Her confessor hath been with her grace this morning, and hath done all that to his office appertaineth, and even now is preparing to minister to her grace the sacrament of unction. At Hampton Court, this Wednesday morning, at eight of the clock." (TURNER's *Life of Henry VIII.*)

